# Adolescents with Chronic Illnesses: School Absenteeism, Perceived Peer Aggression, and Loneliness

**DOI:** 10.1100/tsw.2005.68

**Published:** 2005-07-19

**Authors:** Rosalyn H. Shute, Christine Walsh

**Affiliations:** ^1^ Discipline of Psychology, School of Behavioural and Social Sciences and Humanities, University of Ballarat, P.O. Box 663, Ballarat, Victoria 3353, Australia; ^2^ School of Psychology, Flinders University, GPO Box 2100 Adelaide, South Australia 5001, Australia; ^3^ Boylan Ward, Women's and Children's Hospital, 72 King William Road, North Adelaide, South Australia 5006, Australia

**Keywords:** absenteeism, adolescence, aggression, chronic illness, peer relationships, Australia

## Abstract

Frequent school absence is often cited as a risk factor for peer relationship problems in youngsters with chronic illnesses, but this assumption has not been subjected to quantitative empirical examination. This issue was examined in the present study by exploring the relationship between school absenteeism, peer aggression, and loneliness in adolescents with chronic illnesses. Forty-one adolescents with chronic illnesses completed a modified version of the Direct and Indirect Aggression Scale and the Asher Loneliness Scale. Details of school absences and hospitalizations were obtained from parents and school and hospital records. No evidence was found to support the notion that peer aggression and loneliness are related to absenteeism, but social aggression (for both boys and girls) and verbal aggression (more markedly for girls) were associated with loneliness. Of the group, 19% reported experiencing verbal aggression and 12% social aggression at least weekly; informal qualitative data suggesting that such aggression is often related to limited sporting ability and appearance. Interventions at both the individual and school community level are warranted.

### INTRODUCTION

Significant numbers of children and adolescents in today's industrialized countries are living with chronic illness[1,2]. Although many are well adjusted, as a group, they are at increased risk of psychosocial problems [e.g., 3]. Peer relationships can present difficulties with these adolescents reporting their two most common stressors as "fear of negative evaluations from others" and "fights with or rejection from a friend" [4, p. 288]. It is often suggested that school absenteeism is a risk factor for peer relationship problems in this population [e.g., 4, 5], but this remains to be subjected to quantitative empirical examination. This study provides information about the relationships between loneliness, level of aggression from peers, and frequency of absence in adolescents with chronic illnesses.

Relatively few studies have addressed the issue of how much time students with chronic illnesses do, in fact, lose from school. Cook, Schaller, and Krischer (cited in [1]) found that 5% of children with chronic illness had perfect school attendance, 10% missed 3 months, and some as much as 6 months. There are large individual differences in frequency of absence[6]. Teachers rate children missing 30 days or more as educationally disadvantaged and showing social and emotional difficulties[7]. Previous research does not always make clear whether researchers asked for total days away or days away for the chronic illness; if the latter, absence would be underestimated. Furthermore, the use of parental estimates has been criticized as possibly leading to underestimates of absence[8]. The present study was designed to address these issues.

Loneliness has been defined as "an enduring condition of emotional distress that arises when a person feels estranged from, misunderstood, or rejected by others and/or lacks appropriate social partners for desired activities; particularly activities that provide a sense of social integration and opportunities for emotional intimacy" [9, p. 2]. Fears of loneliness and isolation are expressed by children with chronic illness[1]. These fears are associated with low self-esteem and feelings of being devalued that are increased when the child is absent from school. Several studies indicate that children with chronic, life-threatening illnesses are lonelier than their peers [e.g., 10]. Those children with moderate to severe asthma who experience more hospital admissions report feeling lonelier[5]. By contrast, children with cancer have been found to be similar to matched controls in loneliness, self-concept, and peer acceptance, although perceived by teachers as more socially isolated and having poorer social skills[11]. Overall, chronic illness seems to increase the risk of loneliness. This may become more of an issue in the teens, as adolescents perceive themselves as lonelier than do younger children[9]. The present study aimed to examine whether absenteeism is predictive of loneliness specifically.

While frequent absence may directly cause loneliness by deprivation of rewarding peer-related activities during the period of absence, it may also damage peer relationships as a result of rejection on return to school. The young person is faced with the task of trying to break back into peer groups[12], therefore, social (or indirect) aggression may be a particular issue; this involves deliberate exclusion from group activities and behaviors such as ignoring and spreading nasty rumors [e.g., 13]. It can cause distress to the point of suicidal thoughts and leaving school[14]. Such behavior is more common in the teenage years, and while it happens more frequently among girls [e.g., 15], it predicts symptoms of post-traumatic stress in both boys and girls[16]. This study, therefore, aimed to examine whether peer aggression partially mediates the relationship between absenteeism and loneliness.

While it is generally implied that absenteeism is a *cause* of peer relationship difficulties, it also needs to be acknowledged that it may sometimes *result* from them, as among the general population of school students[17]. Some children hospitalized with asthma are identified as hospital overusers who find relationships with their fellow patients very rewarding[10]. It is, therefore, possible that some with chronic illnesses who have peer relationships problems at school remain at home or in hospital more often, which might lead to a cycle of further peer rejection and absence[18].

To summarize, it was expected that absenteeism, peer aggression, and loneliness would be positively intercorrelated, with aggression partially mediating the relationship between absenteeism and loneliness. Furthermore, in line with previous research[13,15,19], boys were expected to report more direct (physical and verbal) aggression and girls more social aggression from their school peers.

### METHODS

#### Participants

Adolescents with a range of chronic illnesses were included, given evidence that the psychological impact of various conditions is similar [e.g., 20]. However, those with significant visible physical differences

(such as hair loss or limb abnormalities) were excluded, as the focus of the study was on peer aggression in relation to absence and, given the anticipated sample size, it was desirable not to confound this with an additional factor (physical difference) that can, in itself, cause significant difficulties with peer aggression (such as teasing)[21].

A group of 22 girls and 19 boys, aged 11–18 ($M$ = 13.76, $SD$ = 2.09) and their parents participated. All adolescents were patients of the Women's and Children's Hospital in Adelaide, South Australia and had long-term illnesses including cystic fibrosis (7), renal failure (7), diabetes (6), thyrotoxicosis (3), other metabolic disorders (11), asthma (4), juvenile arthritis (1), achalasia (1), and chronic granulomatosis (1).

### Measures

#### School Absence

Data were collected towards the end of the school year. Parents/caregivers were asked to complete a questionnaire indicating the number of days their adolescent had been absent from school during the year for various reasons, including hospitalizations, clinic appointments, staying at home because of the chronic condition, or home for other medical reasons. Parental estimates were cross-checked against school and hospital records.

#### Perceived Aggression

Adolescents completed a modified version of the Direct and Indirect Aggression Scale (DIAS)[13]. This comprises 24 aggressive behavior items, the respondent indicating the frequency with which each occurs on a 4-point scale, generally with regard to same-sex peers. The questionnaire includes the following 3 subscales.

1. Direct physical aggression, e.g., hitting, kicking, taking things
2. Direct verbal aggression, e.g., yelling, insulting, name calling
3. Indirect aggression, e.g., exclusion from the group, ignoring, gossiping

Bjorkqvist et al.[13] developed these subscales using factor and item analysis over a number of studies and reported Cronbach's alphas for each subscale ranging from 0.80–0.94 for most samples. Owens[19] modified the questionnaire to meet South Australian ethics committee standards, whereby respondents could only be asked to provide general estimates of the frequency of such behaviors within their year level, rather than rating individual peers. His findings supported the reliability of the subscales in this format. For the present study, the format was further modified to ask respondents to estimate the frequency of aggression they had personally experienced from same-sex peers over the last four school terms (the academic year). Scoring was as follows: 0 = Never; 1 = Seldom (once a term or less); 2 = Sometimes (every 2–3 weeks); 3 = Often (once or twice a week); 4 = Very Often (almost every day). Cronbach's alphas for perceived physical, verbal, and indirect aggression were 0.85, 0.75, and 0.94, respectively, indicating good reliability.

#### Loneliness

Loneliness was assessed using the Asher, Hymel, and Renshaw Loneliness Scale[22]. Two questions were slightly modified to remove references to "children", as young people aged 11–18 do not identify themselves as such. Adolescents were asked to respond to 24 questions (16 primary items and 8 filler items) by indicating how much each item was true for them on a 5-point scale. Asher et al. reported that the scale was internally consistent and reliable with Cronbach's alpha = 0.90 and 0.83, respectively. Cronbach's alpha for the present sample was also very high (0.93).

### Procedure

The study was approved by the university and hospital ethics committees. Participants were recruited through the hospital outpatient clinics. Direct access to participants was not permitted, with consultants required to make the initial contact. Most data were collected in participants' homes, although the questionnaires of four inpatients were completed in the adolescent ward. Participants were informed that their involvement was voluntary and that their responses would be treated anonymously.

As well as completing the absenteeism questionnaire, parents were asked to sign the consent form for their child's participation and to give written permission for the researcher to obtain school attendance and hospitalization records. All parents consented to the release of information from the hospital. Two did not sign the school consent because the school did not know about the illness. The adolescent questionnaires were administered individually, with the researcher (CW) on hand to answer any queries and debrief. Each session lasted between 30–45 min. All participants were asked to write any comments they considered useful about either the questionnaires or the issues raised and were provided with the opportunity to discuss any matters that arose. While these qualitative comments were not subjected to systematic analysis, they provide some suggestive insights and are summarized in the Discussion.

## RESULTS

### Data Screening and Descriptive Statistics

Multiple regression analyses had been planned in order to examine whether aggression was a partial mediator between absenteeism and loneliness, but as many variables were not normally distributed and not significantly improved by transformations, this was not possible. Nonparametric statistics were therefore predominantly used, with medians and interquartile ranges (IQRs) providing the best measures of central tendency and variability, respectively (means and standard deviations are also provided in the tables). Descriptive statistics for key variables are presented in Table 1.

The medians and IQRs of days absent taken from school records ($Mdn$ = 12, $IQR$ = 27) and parental reports ($Mdn$ = 15, $IQR$ = 25) suggest that parents were quite accurate. A Wilcoxon signed ranks test indicated no significant difference ($z = –1.15$, $p > 0.5$). Furthermore, the two measures were highly correlated ($r_s$ [33] = 0.84, $p < 0.001$). School records were used for further analyses, which considered total days absent. Parental reports indicated that clinic appointments and other illnesses were the most common reasons for school absence. Hospital records showed that 18 participants had no hospital admissions for the year during school time, 7 had 1, 4 had 2, 6 had 3, 1 had 4, and 1 had 5 (missing data, 4). The total number of days in hospital ranged from 1–192 (Table 1).

Medians and IQRs suggest that, on the whole, the adolescents did not perceive themselves as subjected to substantial amounts of aggression (Table 1). However, there was a large variation, with some reporting no aggression and others moderate to high levels for indirect and verbal subscales. Some, 19%, reported experiencing verbal aggression and 12% indirect aggression at least weekly.

The potential maximum loneliness score was 120. Medians and IQRs, therefore, suggest that most participants did not experience high levels of loneliness (Table 1). The scores were quite comparable with those found by Asher et al. who reported a range of 16–79 with a mean of 32.51[22], while in the present study, there was a range of 16–66 with a mean of 31.61.

**TABLE 1**
**Mean and Median Scores, Standard Deviations, and Interquartile Ranges of Key Variables**

| | Mean (SD) | Median (IQR) | Min. | Max. |
|---|---|---|---|---|
| **For total sample** | | | | |
| Days absent (school records) | 26.70 (32.01) | 12.0 (27.00) | 3.0 | 132.00 |
| Admissions to hospital | 1.10 (1.40) | 1.0 (2.00) | 1.0 | 5.00 |
| Days in hospital | 14.30 (34.35) | 1.0 (14.25) | 1.0 | 192.00 |
| Mean physical aggression | 0.46 (0.62) | 0.29 (0.86) | 0 | 2.57 |
| Mean verbal aggression | 1.07 (0.97) | 1.0 (1.10) | 0 | 3.40 |
| Mean indirect aggression | 0.89 (0.90) | 0.5 (1.11) | 0 | 3.50 |
| Loneliness | 31.61 (13.68) | 26.0 (20.00) | 16 | 66.00 |
| **For boys and girls separately** | | | | |
| Days absent | | | | |
| Girls | 22.60 (21.20) | 12.50 (24.0) | 3.0 | 77.50 |
| Boys | 31.94 (41.11) | 10.00 (42.0) | 3.0 | 192.00 |
| Physical aggression | | | | |
| Girls | 0.29 (0.56) | 0.07 (0.32) | 0 | 2.43 |
| Boys | 0.67 (0.64) | 0.57 (0.57) | 0 | 2.57 |
| Verbal aggression | | | | |
| Girls | 0.99 (0.98) | 0.60 (1.20) | 0 | 3.20 |
| Boys | 1.16 (0.97) | 1.00 (1.00) | 0 | 3.40 |
| Indirect aggression | | | | |
| Girls | 1.13 (0.89) | 1.04 (1.60) | 0 | 3.25 |
| Boys | 0.61 (0.84) | 0.33 (0.66) | 0 | 3.50 |
| Loneliness | | | | |
| Girls | 33.70 (15.71) | 28.50 (27.30) | 17.0 | 86.00 |
| Boys | 29.42 (11.16) | 25.00 (17.00) | 16.0 | 59.00 |

For perceived physical aggression, 50% of girls reported it as never occurring compared with 16% of boys. For perceived verbal aggression, both boys (21%) and girls (18%) reported that it sometimes or often happened to them. However, for perceived indirect aggression, 32% of girls reported that it happened sometimes or often, compared with only 11% of boys.

### School Absenteeism in Relation to Perceived Aggression and Loneliness

There was no significant relationship between school absenteeism and physical, verbal, or indirect aggression (Table 2). There was also no significant relationship between the number of hospital admissions and any type of aggression. Similarly, loneliness was not significantly correlated with absenteeism or hospitalizations, either for the entire sample (Table 2) or for boys and girls considered separately (Table 3).

### Perceived Aggression in Relation to Loneliness

As predicted, there was a significant positive relationship between perceived verbal aggression and loneliness and perceived indirect aggression and loneliness (Table 2). However, no significant relationship was identified between perceived physical aggression and loneliness.

**TABLE 2**
**Spearman's Rank Order Correlations Between Demographic, Aggression, and Loneliness Variables**

| | 1. | 2. | 3. | 4. | 5. |
|---|---|---|---|---|---|
| 1. Days absent (from school records) | — | | | | |
| 2. Admissions to hospital | 0.561*** | — | | | |
| 3. Physical aggression | –0.222 | –0.017 | — | | |
| 4. Verbal aggression | –0.235 | –0.063 | 0.442** | — | |
| 5. Indirect aggression | –0.021 | –0.138 | 0.286 | 0.561*** | |
| 6. Loneliness | 0.090 | –0.078 | 0.218 | 0.462** | 0.630*** |

**$p$ <0.01; ***$p$ < 0.001.

*Note:* Correlations involving demographic variables used two tailed test of significance (n = 41).

**TABLE 3**
**Spearman's Rank Order Correlations Between Aggression, Absenteeism, and Loneliness, for Girls and Boys Separately**

| | Physical Aggression | Verbal Aggression | Indirect Aggression |
|---|---|---|---|
| Girls | | | |
| Days absent from school | –0.13 | –0.06 | 0.14 |
| Loneliness | 0.11 | 0.63** | 0.74*** |
| Boys | | | |
| Days absent from school | –0.24 | –0.32 | –0.16 |
| Loneliness | 0.42 | 0.35 | 0.51* |

*$p$ < 0.05; **$p$ < 0.01; ***$p$ < 0.001; (two-tailed significance).

Although no hypotheses were made regarding gender differences in relation to loneliness, these correlations were computed separately for boys and girls. As shown in Table 3, indirect aggression was significantly correlated with loneliness for both boys and girls. Verbal aggression was positively, but not significantly, correlated with loneliness for boys alone, but it was for girls.

### Gender in Relation to Absenteeism and Aggression

A Wilcoxon signed ranks tests indicated that there was no significant difference for gender in days absent ($z$ = –0.460, $p$ > 0.05), although the range was greater for boys (Table 1). Absenteeism was not significantly correlated with any type of aggression for either gender (Table 3).

A Wilcoxon signed ranks test supported the predicted gender difference in indirect aggression ($z$ = –2.17, $p$ < 0.05) with girls (*Mdn* 1.04, *IQR* 1.6) reporting more indirect aggression than boys (*Mdn* 0.33, *IQR* 0.66) (Table 2). A Wilcoxon also supported the prediction that for physical aggression, boys (*Mdn* 0.57, *IQR* 0.57) would report more than girls (*Mdn* 0.071, *IQR* 32) ($z$ = –2.83, $p$ < 0.01). However, there was no significant difference ($z$ = –0.80) between boys (*Mdn* 1, *IQR* 1) and girls (*Mdn* 0.6, *IQR* 1.2) for verbal aggression.

### DISCUSSION

This study set out to test empirically the assumption that absenteeism in adolescents with chronic illnesses would predict loneliness, with peer aggression acting as a partial mediator. While the nature of the data precluded a direct test of the mediation hypothesis, it was clear that this notion was not supportable as there was no significant relationship between absenteeism and either aggression or loneliness. On the other hand, the expected positive relationship between level of aggression and loneliness was found, as were certain predicted gender differences. The study also yielded useful information about the measurement and frequency of absence; some informal qualitative data added further insights.

Those who experienced high levels of indirect aggression reported feeling lonelier, as did those (especially girls) who experienced high levels of verbal aggression (physical aggression was not associated with loneliness). It is important to note that loneliness was not significantly correlated with absenteeism, therefore, the feelings of loneliness cannot be attributed to simply spending more time away from school, but are associated with experiencing social and verbal aggression when attending school. Comments made by the adolescents also supported a relationship between feelings of loneliness and the aggression they experienced, as discussed later.

The lack of a relationship between absence and aggression suggests that there is individual variability in how effectively adolescents are able to maintain or renegotiate their peer relationships during and after periods of absence. Several parents commented that their adolescents worked very hard to ensure that their illness impacted as little as possible on school work and peer relationships. Some adolescents had had 3- to 6-months absences and reported that their peer relationships were unaffected. This may be an accurate perception or reflect a recognized difficulty with self-report measures [e.g., 9,18]. For example, one boy recorded very low DIAS scores and claimed to have lots of friends, but his father mentioned several examples where his son had been very distressed over exclusion from sporting activities and having few same-age friends. Such denial of distress can be an adaptive coping mechanism[21]. Despite the possibility of some underreporting of distress, it is nevertheless very important to use self-reports; the individual is the best judge of subjective feelings of loneliness and social aggression in particular often goes unnoticed by teachers[19].

Although previous researchers have noted the likely impact of absence on peer relationships, none of the adolescents commented negatively about the impact of absenteeism. On the contrary, several indicated a preference for outpatient appointments in school time so that they could miss half or a whole day, which contrasted with parental efforts to minimize school absence.

Whereas Graetz and Shute[5] found that (younger) children with asthma who had more hospital admissions were less preferred as playmates, this study revealed no association between hospitalizations and peer aggression or loneliness. As with absenteeism in general, this may reflect individual variation in coping and possibly underreporting of distress by some participants, as well as the overall low levels of hospitalization. It is important to note that the low admission rates refer only to those during the school term. Some, such as those with cystic fibrosis, usually had planned admissions during school holidays. One adolescent with cystic fibrosis indicated that she enjoyed her hospitalizations; she and her friends with cystic fibrosis were often admitted together and the ward staff allowed her and her friends a lot of time to talk and be “silly”. While this has been identified as an issue for long-term patients in previous research[10], she was the only adolescent who made such comments. However, ward staff indicated that this was more widespread, particularly among those with cystic fibrosis who often displayed behavioral problems. They also indicated that many of these adolescents had refused to participate in the study and believed, based on their long-term association with them, that they did have peer difficulties at school. This informal evidence adds to suggestions that some adolescents with chronic illness develop strong bonds with their ill peers and that this can be perceived by hospital staff as problematic. It also means that the reported levels of aggression and loneliness may be underestimates for the population.

While this study was not aimed at examining differences between those with chronic illnesses and other students, we can observe that Slee reported that 6% of students from the general school population experience physical or psychological bullying 1–2 days per week[17]. In this study, 19% reported verbal aggression and 12% indirect aggression with this kind of frequency. While the present variables and those in the Slee study are not directly equivalent, our findings do indicate a concerningly high percentage of lonely adolescents with chronic illnesses who are subjected to verbal and indirect aggression on at least a weekly basis.

Physical and verbal aggression are generally more common in boys, while social aggression is more common in girls[13,19]. These gender differences were reflected in this sample for physical and social aggression, but there was no difference in verbal aggression, in keeping with Owens' finding with a similarly aged Australian sample[19]. Physical aggression was infrequent, which may reflect success in school antibullying programs; it was not associated with loneliness.

By contrast, both social and verbal aggression were significantly associated with loneliness. The fact that social aggression, although more common in girls, was associated with distress for both genders, is consistent with the finding that experiencing this type of aggression places both boys and girls at risk for symptoms of post-traumatic stress[16]. In addition, in this sample verbal aggression was associated with loneliness, but more strongly for the girls, indicating that experiences such as being called names, insulted, and teased is especially socially isolating for girls. Although those with obvious physical differences were excluded from the study, several adolescents said that their illness did affect their physical appearance at times and they were then subjected to severe verbal taunts and peer group exclusion. For example, one girl said how lonely she was because of her illness and its treatment, which temporarily increased her weight. She had found a caricature of her on a wall at school calling her "the steroid junkie". This resonates with findings that adolescent girls often target others on the basis of appearance, as well as with other findings that show obesity to be stigmatizing [e.g., 23].

Others reported that rejection was due to their inability to play sport. Boys, girls, and their parents commented on the impact of the illness on coordination and endurance; despite willingness to participate, their limitations led to their not being chosen for teams and called names when they played poorly. Difficulty with sports participation has been noted previously for those with chronic illnesses [e.g., 24] and, in the present study, came through as a much more important theme affecting peer relationships than did school absence.

A previous finding of parents' underreporting of school absence[8] was not replicated. Perhaps these parents were particularly vigilant because of hospital education about the school impact of a chronic illness and the increasing emphasis on school achievement. Accuracy may also have been promoted by the questionnaire format, which divided absenteeism into categories, and this method is recommended for future studies.

Overall, adolescents spent a quarter of their time absent attending clinics, in line with previous reports that these contribute substantially to school absence. Although not a focus of this study, a lack of support from schools for the maintenance of academic progress during lengthier absences was consistently noted by parents. This issue has been raised previously[1]; those with prolonged absence need to have a clear plan for school reintegration, with adequate consultation between the health and education systems[1,25].

Low response rates from some of the clinics resulted from the gatekeeping role of consultants as well as involvement of two of the illness groups in other studies. Also, as some with peer problems seem not to have participated, the sample may have been biased toward those who coped better with absence and had better peer relationships. Furthermore, several parents avoided involvement in the project because their adolescents were indeed having peer relationship problems and the parents believed participation would cause distress. Such bias is inevitable with voluntary participation and it seems likely that the level of clinical need is even greater than identified in this sample, especially given the additional likelihood of underreporting of distress by some participants.

It is clear from this study that a significant minority of adolescents with chronic illnesses experience indirect and verbal aggression from their school peers, often centering on illness-related issues, such as appearance and sporting ability. Peer harassment in schools is something that should be addressed for the sake of all young people, but it is especially important to consider interventions for those with chronic illnesses, who already have additional life challenges to face. An ongoing issue in the literature on victimization is whether victims attract negative peer behaviors, for example, through a lack of social skill, or whether receiving negative peer behaviors cause problems with social adaptation, such as loneliness. While this cannot be determined in a cross-sectional study such as this, the general literature suggests that both are at play and it seems likely that the same is true of the chronically ill population. These children need to be provided with strategies for dealing with peer comments and rejection early and, as they enter their teens, these issues should be readdressed in a developmentally appropriate way. Kish and Lansdown, with regard to interventions for children with craniofacial anomalies, note that social skills interventions for such groups are not premised on a lack of social skills, but on helping those at risk of negative social reactions to develop coping strategies[21]. However, it is not appropriate to place all the burden of dealing with this issue on the young people themselves and broader action, such as exploring ways of incorporating social aggression into school antiharassment policies and practices, may help to provide a school context in which such aggression is less likely.

### ACKNOWLEDGMENTS

We are grateful to the participating young people, parents, and schools.

## References

1. Sexson, S.B. and Madan-Swain, A. (1993) School re-entry for the child with a chronic illness. *J. Learn. Disabil.* **26(2),** 115–125.
2. Shute, R.H. (1998) Peer relationships of children with chronic illness. In *Children's Peer Relations: Current and Future Directions.* Slee, P. and Rigby, K., Eds. Routledge, London. pp. 134–143.
3. Seigel, W.M., Golden, N.H., Gough, J.W, et al. (1990) Depression, self-esteem, and life events in adolescents with chronic diseases. *J. Adolesc. Health Care* **11,** 501–504.
4. La Greca, A.M. (1990) Social consequences of pediatric conditions: fertile area for future investigation and intervention? *J. Pediatr. Psychol.* **15(3),** 285–307.
5. Graetz, B. and Shute, R. (1995) Assessment of peer relationships in children with asthma. *J. Pediatr. Psychol.* **20,** 205–216.
6. Newacheck, P.W. and Taylor, W.R. (1992) Childhood chronic illness: prevalence, severity, and impact. *Am. J. Public Health* **82(3),** 364–371.
7. Anderson, H.R., Bailey, P.A., Cooper, J.S., et al. (1983) Morbidity and school absence caused by asthma and wheezing illness. *Arch. Disabled Child* **58,** 777–784.
8. Hill, R.A., Standen, P.J., and Tattersfield, E. (1989) Asthma, wheezing, and school absence in primary schools. *Arch. Dis. Child.* **64,** 246–251.
9. Freidman, G., Florian, V., and Zernitsky-Shurka, E. (1989) The experience of loneliness among young adult cancer patients. *J. Psychosoc. Oncol.* **7(3),** 1–15.
10. Creer, T.L. (1993) Psychological impact of childhood asthma. In *Childhood Asthma: Pathophysiology and Treatment.* Tinkelman, D.G. and Naspitz, C.K., Eds. Marcel Dekker, New York. pp. 553–589.
11. Noll, R.B., LeRoy, S., Bukowski, W.M., et al. (1991) Peer relationships and adjustment in children with cancer. *J. Pediatr. Psychol.* **16(3),** 307–326.
12. Ambert, A. (1984) A qualitative study of peer abuse and its effects: theoretical and empirical implications. *J. Marriage Fam.* **56,** 119–130.
13. Bjorkqvist, K., Lagerspetz., K.M.J., and Østerman, K. (1992) The Direct and Indirect Aggression Scales. Abo Akademi University, Department of Social Sciences, Vasa, Finland.
14. Owens, L., Slee, P., and Shute, R. (2000) "It hurts a hell of a lot …" The effects of indirect aggression on teenage girls. *Int. J. School Psychol.* **21(4),** 359–376.
15. Owens, L.D. and MacMullin, C.E. (1995) Gender differences in aggression in children and adolescents in South Australian schools. *Int. J. Adolesc. Youth* **6,** 211–235.
16. Mynard, H., Joseph, S., and Alexander, J. (2000) Peer-victimisation and posttraumatic stress in adolescents. *Personal. Individ. Diff.* **29,** 815–821.
17. Slee, P.T. (1995). Bullying: health concerns of Australian secondary school students. *Int. J. Adolesc. Youth* **5,** 1–10.
18. Renshaw, P.D. and Brown, P.J. (1993) Loneliness in middle childhood: concurrent and longitudinal predictors. *Child Dev.* **64,** 1271–1284.
19. Owens, L.D. (1996) Sticks and stones and sugar and spice: girls' and boys' aggression in schools. *Aust. J. Guidance Counselling* **6,** 45–55.

20. Fischer, T.J. (1993) Problems in the diagnosis and management of the asthmatic adolescent. In *Childhood Asthma: Pathophysiology and Treatment.* Tinkelman, D.G. and Naspitz, C.K., Eds. Marcel Dekker, New York. pp. 413–428.
21. Kish, V. and Lansdown, R. (2000) Meeting the psychosocial impact of facial disfigurement: developing a clinical service for children and families. *Clin. Child Psychol. Psychiatry* **5(4),** 497–512.
22. Asher, S.R., Hymel, S., and Renshaw, P.D. (1984) Loneliness in children. *Child Dev.* **55,** 1456–1464.
23. Bell, S.K. and Morgan, S.B. (2000) Children's attitudes and behavioral intentions toward a peer presented as obese: does a medical explanation for the obesity make a difference? *J. Pediatr. Psychol.* **25(3),** 137–145.
24. Christie, M.J., French, D., and Weatherstone, A. (1994) The patient's perception of chronic disease and its management: psychosomatics, holism and quality of life in the contemporary management of childhood asthma. *Psychother. Psychosom.* **56,** 197–203.
25. Shute, R. (1999) Childhood chronic illness and the school. *Aust. J. Guidance Counselling* **9(1),** 109–121.

### BIOSKETCHES

**Rosalyn Shute, BSc (Hons), PhD,** is Professor and Director of Psychology at the University of Ballarat, Victoria, Australia, where her major teaching commitments are at the postgraduate level in clinical and health psychology. Her numerous publications include books, book chapters, and refereed journal articles on various aspects of child and adolescent health and development, including chronic illness, vision care, peer relationships, social support, and aggression. E-mail: r.shute@ballarat.edu.au. Website: http://www.ballarat.edu.au/ard/bssh/staff/psychstaff.html

**Christine Walsh, RMHN, M. Psych. (Clin),** currently works in the area of child and adolescent mental health as a private psychologist in Adelaide, South Australia, and as the Nursing Unit Head of a statewide acute child and adolescent mental health inpatient unit in a metropolitan hospital. She was awarded the Nursing Excellence Award in Metropolitan Acute Care in 2005 for recognition of her work in providing appropriate and innovative care to young people and promoting nurses in the speciality of mental health.
